# Successful management of a pregnant woman with Kasabach–Merritt syndrome and preeclampsia: A case report

**DOI:** 10.1097/MD.0000000000021198

**Published:** 2020-07-10

**Authors:** Yi Yang, Zhiheng Guo, Zhenpeng Wang, Lili Luo, Ying Chen

**Affiliations:** a Center of Reproductive Medicine and Center of Prenatal Diagnosis; b Department of Obstetrics; c Department of Gynecologic Oncologic, The First Hospital of Jilin University, Changchun, Jilin, China.

**Keywords:** cesarean section, incision bleeding, Kasabach-Merritt Syndrome, postpartum hemorrhage, preeclampsia

## Abstract

**Introduction:**

Kasabach-Merritt Syndrome (KMS) is an extremely rare disease in adults, which lead to consumptive coagulopathy characterized by severe hypofibrinogenemia and thrombocytopenia.

**Patient concerns:**

a 25-year-old Chinese pregnant women complicated by preeclampsia and KMS presented with refractory postpartum hemorrhage and incision bleeding after cesarean section.

**Diagnosis:**

The diagnosis of KMS was made based on clinical manifestation of Kaposiform Hemangioendothelioma, severe hypofibrinogenemia and thrombocytopenia.

**Interventions:**

After a poor response to massive blood products transfusion for 1 week, corticosteroid treatment was initiated for 3 days.

**Outcomes:**

The patient reached a normal platelet count and a mild anemia within 4 weeks. Two months later, all laboratory values had returned to normal, and the incision was healing well.

**Conclusion:**

Pregnancy complicated by preeclampsia and surgery may have contributions for the development of Kasabach–Merritt syndrome. Corticosteroid is indicated in the episode of acute Kasabach–Merritt syndrome after the failure of massive blood transfusion.

## 1 Introduction

Tufted Angioma (TA) and Kaposiform Hemangioendothelioma (KHE) are 2 kinds of rare vascular tumors which mostly occur in infancy and early childhood. The most serious complication of KHE and TA is Kasabach-Merritt Syndrome (KMS), a consumptive coagulopathy characterized by severe hypofibrinogenemia and thrombocytopenia. Macroscopically KMS is the locally aggressive abundant vascular structures that infiltrate the soft tissues with consumptive disseminated intravascular coagulation (DIC). The incidence of KHE is estimated to be 0.07/100, 000 based on observed cases at the Vascular Anomalies Center of Children's Hospital Boston for KHE between 1991 and 2009,^[1]^ and the mortality rate is as high as 30% to 40% as a result of irrepressible bleeding.^[2]^ There is no consensus or guideline for the pregnant women with KMS because of its rarity and the lack of prospective trials. We report a 25-year-old Chinese pregnant women complicated by preeclampsia and KMS involving right hand, right arm and the posterior aspect of the right shoulder presented with refractory postpartum hemorrhage and incision bleeding after cesarean section. To our knowledge, it is the first instance reported in which the syndrome has been associated with pregnancy and preeclampsia. We aim to provide new information about this entity and contributions to better treatment.

## 2 Case report

Publication of this case was approved by the Ethics Committee of the First Hospital of Jilin University. Informed written consent was obtained from the patient for publication of this case report and accompanying images.

**Figure 1:**
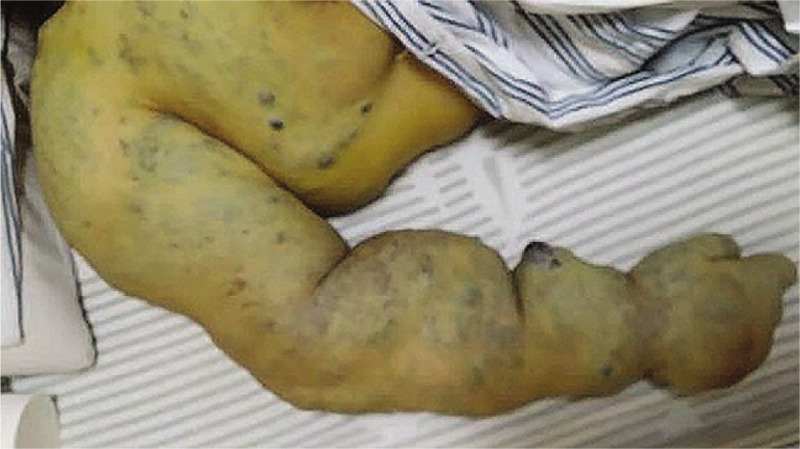
Jaundice in the patient's skin and dark purple lumps surrounding the right arm.

A 25-year-old primigravida, who undertook cesarean section due to preeclampsia 6 days before in a local hospital, was transferred to our hospital for refractory postpartum hemorrhage and incision bleeding after the operation. On physical examination, she displayed a large area of subcutaneous bleeding around the transverse incision, extending to umbilicus and bilateral axillary midline, which was 5 centimeters higher than normal abdominal wall, and active bleeding could be observed along the incision line. In addition a giant hemangiomata,15 cm in size, located over the right hand, right arm and the posterior aspect of the right shoulder (Fig. 1). This hemangiomas diagnosed by MIR has been present since birth without receiving systemic treatment. Hypofibrinogenemia and hypertension had been found before operation. The patient was in a poor condition with blood pressure (BP) of 161/103mm Hg and heart rate (HR) of 100. Her abdomen CT scan revealed the presence of a heterogeneous mass with a dimension of 14.5 cm × 4.1 cm under the incision. Laboratory investigations revealed hemoglobin, 59 g/L, platelet count,78x10^9^ /L, prothrombin time,12.5 s (control: 9–13 s), activated partial thromboplastin time, 28.3 second (control: 20–40 second), international normalized ratio,1.03 (control:0.80–1.20), prothrombin activity,55% (control: 80%–120%) serum fibrinogen,1.00 g/L (control: 2–4 g/L), D-dimer,54690ug/L, serum total bilirubin,49.7uoml/L, and normal liver and renal functions. The diagnosis of KHE associated with KMS was considered. Pressure hemostasis was applied to deal with incision bleeding. Given the patient's low platelet count and laboratory signs of consumptive coagulopathy, Transfusion of red blood cell, platelet, and fibrinogen was carried out at the first day of admission. Anaemia, hypofibrinogenemia, and thrombocytopenia were transiently improved but the effect did not persist. In spite of continuous transfusion of fibrinogen and ingredient blood for 1 week (20U red blood cell, 10 U platelet, 20 U cryoprecipitate in total), she remained anemic, thrombocytopenic, and coagulopathic. Platelets counting was between 58 and 90 × 10^9^/L, plasma fibrinogen concentration fluctuated between 0.41 - 1.53 g/L, and hemoglobin,65 g/L,. Because of her refractory coagulopathy, the patient was started on corticosteroid (Dexamethasone, with a dose of 20 mg per day for 3 days). Fibrinogen was continued every day to stable the low level state, but other blood products transfusion was stopped. Platelet count began to recover immediately after corticosteroid was administered. Her platelet counts increased to 141 × 10^9^/L and HGB to 100 g/L without the blood products transfusion, and coagulation profile was normal except fibrinogen on postoperative day 38. Incision bleeding decrease gradually and fresh granulation tissue grew up with the adjunctive therapy of infrared irradiation physiotherapy. She was then transferred to the local hospital for further treatment. Two months later, all laboratory values had returned to normal, and the incision was healing well.

## 3 Discussion

TA and KHE are thought to be part of the same neoplastic spectrum for owning collaborative histopathologic and clinical features. The most serious complication of both vascular tumors is KMS. The etiology and pathogenesis of the syndrome is currently unclear, which is characterized by profound thrombocytopenia and consumptive coagulopathy. The pathophysiology of KMS is theorized that platelets become trapped in the lesion, leading to platelet activation and fibrinogen consumption. One hypothesis was raised that abnormal vascular structure, turbulent flow, and sheer stress in proliferation and activation would increase adhesion of platelets, and their secondary aggregation and activation. Once the process occurs, subsequent consumption of fibrinogen and coagulation factors will begin, followed by fibrinolysis.^[3]^

The syndrome typically manifests at infancy or early childhood, and only few cases with onset in adult have been reported. To our knowledge, there are only 3 cases reporting KMS associated with pregnancy in the literature.^[4]^ Lee & Kirk^[5]^ firstly reported the syndrome occurring in a pregnant woman with congenital haemangiomata in 1967. Neubert et al^[6]^ reported a 22-year-old primigravida, who had multiple hemangiomas in the right lower quadrant of the abdomen, developed KMS after cesarean delivery. Girija Singh et al^[7]^ described the syndrome occurred in a woman who have multiple hemangiomas, distributed over the right temporal region, right side of the chin, right side of the nose, left upper part of the abdominal wall, and anterior aspect of the left leg in 2 successive pregnancies. It has been suggested that the syndrome may occur as a result of hormonal alterations and increase in blood volume during pregnancy. In our case, surgery and pregnancy hypertension may have contributions to the episode of the syndrome.

Though standardized treatment protocols have not been established, several treatments have been proposed. Vincristine has been the most widely used first-line treatment for KMS,^[8]^ which can effectively increase the patient's platelet count and significantly reduce the lesion. Other medicine treatments included cyclophosphamide, actinomycin D, ticlopidine, aspirin, and methotrexate,^[9–12]^ however the pharmacologic therapy guideline and consensus has not been established. In many cases, multiple agents are selected in sequence or combination since no single agent is presumed to be the most effective.

Surgical management,^[12]^ endovascular intervention and radiation^[12]^ are 3 definitive non-pharmaceutical cures for KHE. Radiotherapy has been proven to be effective, especially for KMS confined to a single lesion.^[7]^ The aim of all 3 treatments is to reduce or eliminate tumors. The patient reported is a congenital KME patient combined with pregnancy, who failed to receive reasonable treatment at childhood and grew into adulthood with the huge tumor. The tumor invaded the entire hand and right upper limb to the shoulder. During pregnancy, these non-pharmaceutical treatments are obviously inappropriate.

In this case, the cause of incision bleeding and the hematoma around the incision was the consequence of coagulopathy and thrombocytopenia. The patient received massive transfusion of blood products under the premise of anti-infection. Thrombocytopenia and hypofibrinogenemia can not be effectively improved because the huge hemangioma consumed fibrinogen and platelets in extremely fast rate. Transfusion mainly aimed to maintaining the hemoperfusion of vital tissue and organs, even it can only maintain the hemoglobin at a low level. The consumption of platelets and fibrinogen mainly considered as a result of KMS caused by the huge hemangioma and pregnancy hypertention. The decrease of hemoglobin stems from 2 factors: 1 is due to the continuous, slow blood loss of the incision bleeding, the other is deduced that mechanical hemolysis in vivo may be involved.

The mechanism of corticosteriod in controlling thrombocytopenia, coagulopathy, and hemangioma has not been verified, but it play a role in increasing platelet counts and vasoconstriction.^[7]^ Glucocorticoid may deduce hemolysis by stabling membrane and improve anemia. The emphasis of treatment was concentrated on correction of coagulopathy and anemia to promoting incision healing. Successful treatment of the puerpera with KMS suggested that short-course glucocorticoid therapy is likely an effective strategy for the stability of the disease and subsequent treatment.

KMS reports are mostly infants and young children, and rarely seen in adults. Neither single or combination therapy have been found to be effective in a uniform or reproducible manner for KMS. Experiences with pregnant women with KMS are still scarce and further research is needed to obtain better treatment options.

## Author contributions

**Conceptualization:** Ying Chen

**Data acquisition:** Zhiheng guo

**Formal analysis:** Ying Chen, Zhiheng Guo

**Investigation:** Zhenpeng Wang, Yi Yang

**Methodology:** Ying chen, Yi Yang

**Software:** Zhiheng Guo

**Supervision:** Zhenpeng Wang, Lili Luo

**Writing – original draft:** Ying Chen, Zhiheng Guo

**Writing – review & editing:** Yi Yang, Zhiheng Guo
